# The impact of prior exposure to hypoglycaemia on the inflammatory response to a subsequent hypoglycaemic episode

**DOI:** 10.1186/s12933-023-02095-w

**Published:** 2024-02-08

**Authors:** Clementine E. M. Verhulst, Julia I. P. van Heck, Therese W. Fabricius, Rinke Stienstra, Steven Teerenstra, Rory J. McCrimmon, Cees J. Tack, Ulrik Pedersen-Bjergaard, Bastiaan E. de Galan, the Hypo-RESOLVE consortium

**Affiliations:** 1 Department of Internal Medicine, Radboud University Medical Centre, P.O. box 9101, 6500 HB Nijmegen, The Netherlands; 2 Department of Endocrinology and Nephrology, Nordsjællands Hospital, Hillerød, Denmark; 3 Division of Human Nutrition and Health, Wageningen University, Wageningen, The Netherlands; 4 Section Biostatistics, Department for Health Evidence, Radboud Institute for Health Sciences, Radboudumc, Nijmegen, The Netherlands; 5 School of Medicine, University of Dundee, Dundee, Scotland; 6 Department of Clinical Medicine, Faculty of Health and Medical Sciences, University of Copenhagen, Hillerød, Denmark; 7 Department of Internal Medicine, Maastricht University Medical Centre, MUMC+, Maastricht, The Netherlands; 8 CARIM School for Cardiovascular Diseases, Maastricht University, Maastricht, The Netherlands

**Keywords:** Antecedent hypoglycaemia, Clamp, Diabetes, Inflammatory responses, Humans

## Abstract

**Background:**

Hypoglycaemia has been shown to induce a systemic pro-inflammatory response, which may be driven, in part, by the adrenaline response. Prior exposure to hypoglycaemia attenuates counterregulatory hormone responses to subsequent hypoglycaemia, but whether this effect can be extrapolated to the pro-inflammatory response is unclear. Therefore, we investigated the effect of antecedent hypoglycaemia on inflammatory responses to subsequent hypoglycaemia in humans.

**Methods:**

Healthy participants (*n* = 32) were recruited and randomised to two 2-h episodes of either hypoglycaemia or normoglycaemia on day 1, followed by a hyperinsulinaemic hypoglycaemic (2.8 ± 0.1 mmol/L) glucose clamp on day 2. During normoglycaemia and hypoglycaemia, and after 24 h, 72 h and 1 week, blood was drawn to determine circulating immune cell composition, phenotype and function, and 93 circulating inflammatory proteins including hs-CRP.

**Results:**

In the group undergoing antecedent hypoglycaemia, the adrenaline response to next-day hypoglycaemia was lower compared to the control group (1.45 ± 1.24 vs 2.68 ± 1.41 nmol/l). In both groups, day 2 hypoglycaemia increased absolute numbers of circulating immune cells, of which lymphocytes and monocytes remained elevated for the whole week. Also, the proportion of pro-inflammatory CD16^+^-monocytes increased during hypoglycaemia. After ex vivo stimulation, monocytes released more TNF-α and IL-1β, and less IL-10 in response to hypoglycaemia, whereas levels of 19 circulating inflammatory proteins, including hs-CRP, increased for up to 1 week after the hypoglycaemic event. Most of the inflammatory responses were similar in the two groups, except the persistent pro-inflammatory protein changes were partly blunted in the group exposed to antecedent hypoglycaemia. We did not find a correlation between the adrenaline response and the inflammatory responses during hypoglycaemia.

**Conclusion:**

Hypoglycaemia induces an acute and persistent pro-inflammatory response at multiple levels that occurs largely, but not completely, independent of prior exposure to hypoglycaemia.

*Clinical Trial information* Clinicaltrials.gov no. NCT03976271 (registered 5 June 2019).

**Supplementary Information:**

The online version contains supplementary material available at 10.1186/s12933-023-02095-w.

## Introduction

Hypoglycaemia is the most common complication of insulin treatment, reportedly affecting people with type 1 diabetes on a twice weekly basis [1]. We and others have shown that hypoglycaemia induces a pro-inflammatory response, consisting of increased numbers of circulating immune cells, including pro-inflammatory monocytes, increased ex vivo monocyte responsiveness and circulating pro-inflammatory cytokines in people with or without diabetes, an effect that is sustained for a week [2–5].

While hypoglycaemia stimulates the release of several counterregulatory hormones, including adrenaline, to restore normoglycaemia, recurrent hypoglycaemic events attenuate this response [6]. Whether recurrent hypoglycaemia also attenuates the pro-inflammatory response is unclear. Adrenaline may be involved in the inflammatory responses to hypoglycaemia and one study reported reduced inflammatory responses in people with diabetes and attenuated adrenaline responses to hypoglycaemia [4]. Adrenaline administration in healthy individuals has been shown to result in mobilization of leukocytes with cytotoxic effector potential from the marginal pool (vascular epithelium) [7].

Here, we investigated the effect of antecedent hypoglycaemia on pro-inflammatory responses to a subsequent hypoglycaemic event in healthy individuals, using a comprehensive approach that consisted of immune cell composition, phenotype and function, and circulating inflammatory proteins including hs-CRP.

## Research design and methods

### Study design

This was a two-centre study, performed at the Radboud University Medical Center in Nijmegen, The Netherlands and the Nordsjællands University Hospital in Hillerød, Denmark. The study was approved by local institutional review boards and performed according to the principles of the Declaration of Helsinki. All participants gave written informed consent.

#### Participants

This study is part of a larger project (NCT03976271), details of which have been published previously [8, 9] and performed under the Europeaon IMI project HypoResolve (website). For this study, we recruited healthy participants between August 2019 and March 2021. All participants were eligible when they were between 18 and 80 years old, had a BMI of 19–40 kg/m^2^, HbA_1c_ < 42 mmol/mol (6%) and a blood pressure < 140/90 mmHg. Exclusion criteria were the use of any medication (except for oral contraceptives), pregnancy, breastfeeding or unwillingness to undertake measures for birth control, and an infection or vaccination in the previous three months.

#### Study procedure

**Fig. 1:**
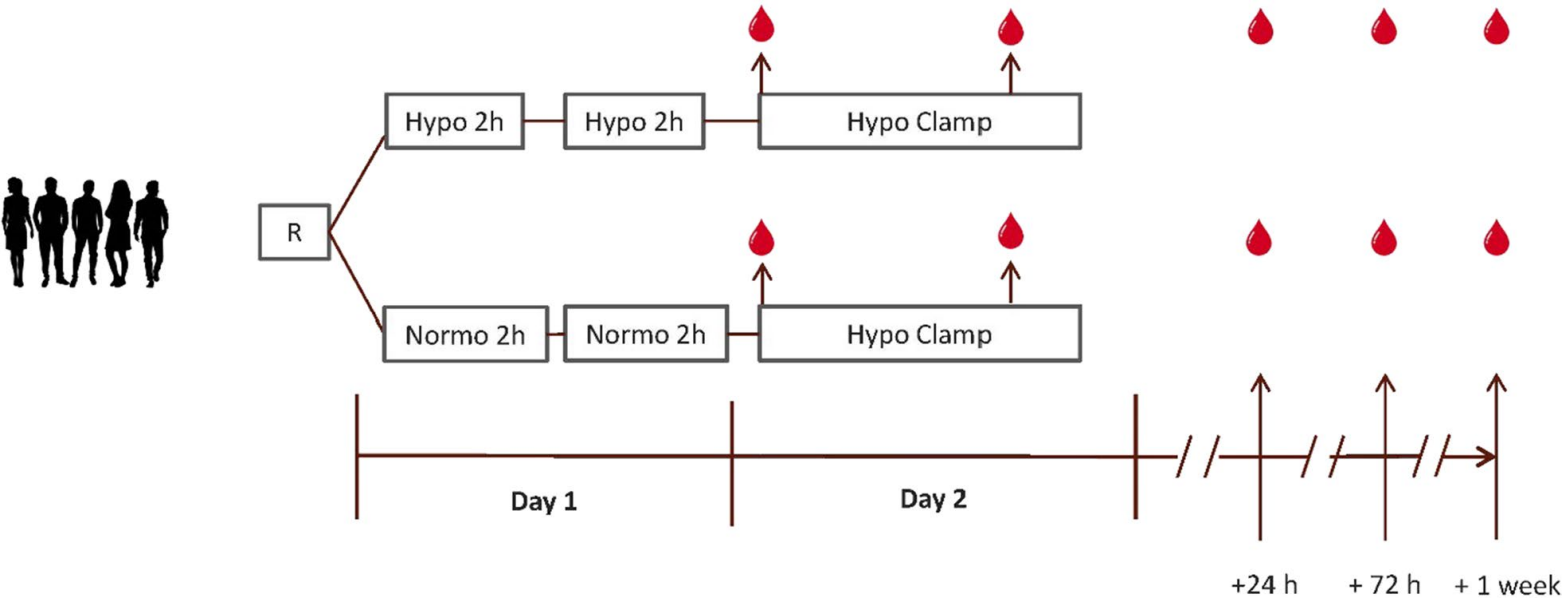
Flowchart study procedure

All eligible study participants were invited for a screening visit, including medical history, standard physical examination and measurement of HbA_1c_ and serum creatinine. After inclusion, participants were randomised to either the antecedent hypoglycaemia (HYPO) study-arm or to the antecedent normoglycaemia (NORMO) study-arm. Participants assigned to HYPO and NORMO study-arms were exposed to two 2-h episodes of hypoglycaemia or normoglycaemia, respectively, on day 1, using the hyperinsulinaemic glucose clamp technique (Fig. 1). The next morning, all participants underwent a hyperinsulinaemic-hypoglycaemic glucose clamp, details of which are described below.

#### Day 1 study protocol

Participants arrived at the research facility in fasting condition between 0700 and 0800 h, having abstained from alcohol, caffeine-containing substances and smoking for at least 24 h and from strenuous exercise for 48 h. Upon arrival, an intravenous catheter was inserted into an antecubital vein of one arm for continuous administration of insulin aspart (Novo Nordisk, Bagsværd, Denmark) at a rate of 1.5 mU kg^−1^ min^−1^ and a variable infusion of glucose 20% (Baxter B.V., Deerfield, IL). In the dorsal vein of the contralateral hand, a second catheter was inserted in retrograde fashion for frequent blood sampling, with the hand placed in a heated box (temperature ~ 55 °C) to arterialise venous blood. Plasma glucose levels were measured (Biosen C-Line; EKF Diagnostics, Cardiff, U.K.) at baseline and at 5–10 min intervals. After obtaining baseline samples, plasma glucose was allowed to fall to 2.8 mmol/L or maintained at normoglycaemia (5.0–5.5 mmol/L) for two 2-h periods, interspersed with a 2-h period of normoglycaemia (total duration, 6 h) in participants randomised to HYPO and NORMO study-arms, respectively. All participants received a snack during the normoglycaemic break. At the end of the clamp, the insulin infusion was stopped, participants received a meal and glucose infusion was increased and then tapered until stable normoglycaemic levels were reached.

#### Day 2 study protocol

On day 2, all participants were requested to return to the research facility between 0700 and 0800 h, in fasting condition similar to that for day 1, to undergo a hyperinsulinaemic normoglycaemic-hypoglycaemic glucose clamp, as described previously [8]. Briefly, using the abovementioned procedures for conducting the clamp, plasma glucose levels were kept at normoglycaemia (5.0 mmol/L) for 30 min, after which these were allowed to drop to 2.8 mmol/L over 20–30 min and maintained at this level for another 60 min. Then, the insulin infusion was stopped, glucose infusion was increased and then tapered until stable normoglycaemic levels were reached.

### Measurements

A modified Edinburgh Hypoglycaemia Score [10] was used to assess the nature and intensity of hypoglycaemic symptoms at baseline (before onset of insulin infusion), during normoglycaemia, and twice during hypoglycaemia on both study days. Autonomic symptoms (sweating, anxious, tingling of hands and feet, palpitations, hunger, trembling and shivers), neuroglycopenic symptoms (feeling warm, confused, inability to concentrate, blurry vision, tiredness, difficulty of speaking, weakness, double vision, dizziness, drowsiness) and general symptoms (headache and nausea) were assessed and ranked from 1 (none) to 7 (severe).

Blood was drawn for measurements of insulin and counterregulatory hormones (glucagon, adrenaline, noradrenaline, cortisol and growth hormone (GH) at baseline, start and at 60 min duration of the first hypoglycaemic phase on day 1, and at baseline, end of normoglycaemia and start and end (60 min) of hypoglycaemia on day 2. In addition, blood was also drawn on day 2 for inflammatory analysis at the end of normoglycaemia and hypoglycaemia and 24 h day, 72 h and 1 week thereafter.

### Laboratory analysis

Serum creatinine was determined with an enzymatic assay on a Cobas 8000 c702 (Roche Diagnostics, Woerden, The Netherlands). HbA_1c_ was assessed by the TOSOH G8 and G11 HPLC-analyser (Sysmex, Etten-Leur, The Netherlands). Plasma adrenaline and noradrenaline were measured by HPLC in combination with fluorometric detection. Plasma insulin was analysed with an in-house radioimmunoassay. The plasma glucagon concentration was measured using radioimmunoassay [11]. Plasma cortisol and GH were determined by a routine analysis method with an electrochemiluminescent immunoassay on a Modular Analytics E170 (Roche Diagnostics, GmbH, Mannheim, Germany).

#### Immune cell number and composition

Immune cell subset numbers were measured on a Sysmex XN-450 and Sysmex XN-9000 (Sysmex). FACS analysis was performed in one of the two participating study sites, because this method is sensitive to confounders when performed at different sites. A total of 50 μl of whole undiluted blood was incubated for a duration of 15 min in the dark at room temperature with the following antibodies: CD16-FITC (dilution 1:20), CD14-PC7 (1:20), CCR2-BV421 (1:20) (BD Biosciences, Vianen, the Netherlands); CD41-PC5.5 (1:20), CD11b-BV785 (1:20) (ITK Diagnostics BV, Uithoorn, the Netherlands); HLA-DR-PE (1:10), CD56-APC (1:10), CD3-APC-750 (1:10), CD45-KO (1:10), CD36-APC-700(1:10) (Beckman Coulter, Woerden, the Netherlands). Subsequently, 1 ml of lysis buffer (BD Pharm Lyse, BD Biosciences) was added, samples were mixed, incubated for another 10 min and then measured on a flow cytometer (Beckman Coulter FC500). To determine the position of analysis gates, single staining and fluorescence-minus-one control stains were used (Additional file 1: Fig. S1). To analyse the FACS data, Kaluza software (Beckman Coulter, Woerden, the Netherlands) was used.

#### Isolation of PBMCs and Monocytes and ex vivo function

Peripheral blood mononuclear cells (PBMCs) were isolated from whole blood using density centrifugation over Ficoll-Paque (GE Healthcare, UK). Monocytes were subsequently isolated from PBMCs using magnetic activated cell sorting (MACS) MicroBeads (Miltenyi Biotec) for CD14 negative selection according to the manufacturer’s instructions. The purity of monocyte isolation was checked using Sysmex XN-450 and XN-9000.

CD14 negative selected human monocytes (100.000 cells/well) were added to flat bottom 96-wells plates and stimulated with RPMI, 20 µg/mL of Pam3Cys (P3C, a TLR-2 agonist) or 20 ng/mL of lipopolysaccharide (LPS, a TLR-4 agonist) for 24 h. Supernatants were collected and stored at − 20 °C until cytokine measurements using ELISA that included tumour necrosis factor-α (TNF-α) (R&D, Minneapolis, Minnesota, USA), interleukin-10 (IL-10) (R&D), interleukin 1β (IL-1β) (R&D) and Interleukin-6 (IL-6) (R&D).

#### Inflammatory markers

Plasma high sensitive C-reactive Protein (hs-CRP) concentrations were assessed by ELISA following manufacturer’s instructions (R&D). Plasma samples were kept at -80^◦^C until measurement and were measured in one batch using timepoints normoglycaemia, hypoglycaemia, 24 h and 1 week. A total of 92 circulating plasma inflammatory proteins were measured using the commercially available Olink Proteomics AB Inflammation Panel (Uppsala, Sweden). Proteins are recognised by antibody pairs coupled to cDNA strands which bind in close proximity and extend by a polymerase reaction [12]. A threshold of 75% was used, and proteins were excluded for analysis when the threshold was not met. All samples passed the quality control performed by Olink Proteomics. Overall, 72 of the 92 (78%) proteins were detected in at least 75% of the plasma samples and included in the analysis.

### Statistical analysis

All normally distributed data are shown as percentages or mean ± SD, unless otherwise indicated. All non-normally distributed data were log transformed before analyses, e.g. the ex vivo cytokine data. The following analyses were performed for all parameters except the Olink data. Independent t-tests were used to compare continuous data between the two participant groups. Repeated measurements data were analysed with mixed models analysis, where the dependent variable was the result of the measured parameter (e.g. immune cell number) of each timepoint and the independent parameter was “time”. Next to “time”, “participant group” was added as independent variable in this analysis to compare serial data between the study-arms. For the Olink data, the following analyses were performed. Visualisation was done using the R programming language and R packages “ggbiplot” and “ggplot2”. To detect proteins that were significantly affected by hypoglycaemia as compared to normoglycaemia, separately in the HYPO and in the NORMO group, a Wilcoxon matched pairs test between the normoglycaemia and each post-hypoglycaemia measurement (24 h, 72 h and 1 week) was performed. Subjects with missing values were excluded from Olink analyses. Spearman or Pearson correlation tests were used to determine the correlation between mean increase in adrenaline during hypoglycaemia and the delta of immune cell numbers, phenotype and function for both study groups together during hypoglycaemia compared to normoglycaemia. Statistical analyses were performed using IBM SPSS Statistics 27 or R Studio (Version 1.4.1717). Alpha was set at 0.05 throughout, unless otherwise stated.

## Results

**Fig. 2:**
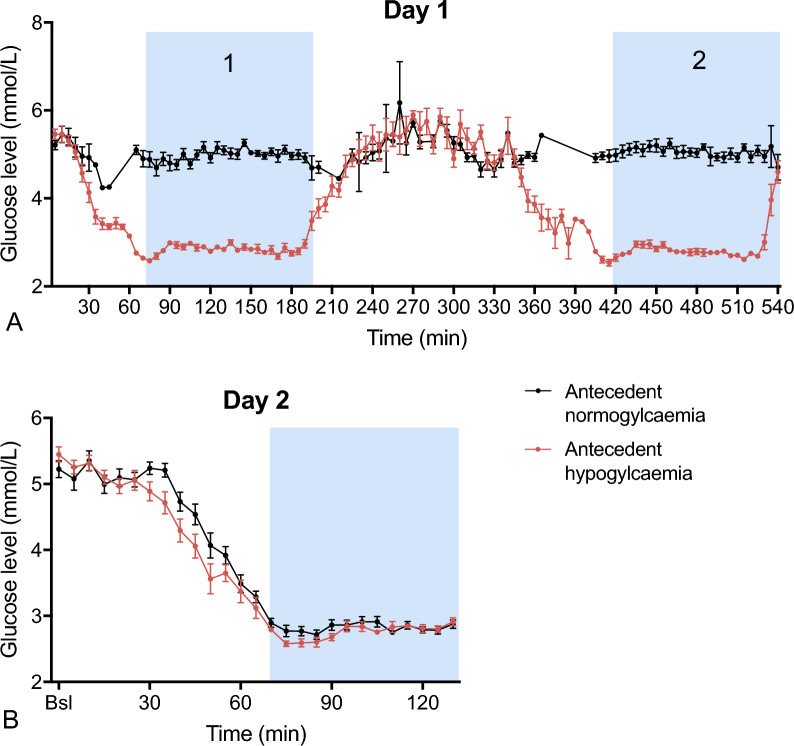
Achieved glucose levels of participants that underwent a recurrent hypoglycaemic event (red) or normoglycaemic event (black) on experimental day 1 (**A**) and, experimental day 2 (**B**)

A total of 32 participants were enrolled and randomised to HYPO or NORMO study-arms. The groups were well matched for age (43.6 ± 17.9 vs. 44.3 ± 18.6 years), sex (7 males and 9 females in both groups) and body-mass index (BMI) (23.6 ± 2.1 vs. 22.6 ± 2.8 kg/m^2^) (Table 1). On experimental day 1, glucose levels averaged 2.83 ± 0.13 mmol/L and 2.75 ± 0.09 mmol/L for the two hypoglycaemic episodes in the HYPO group, and 5.03 ± 0.22 mmol/L and 5.08 ± 0.18 mmol/L for the corresponding normoglycaemic episodes in the NORMO group, respectively (Fig. 2A). On day 2, glucose levels averaged 5.17 ± 0.33 mmol/L and 5.20 ± 0.38 mmol/L (*p* = 0.815) during the normoglycaemic phase and 2.76 ± 0.11 mmol/L and 2.84 ± 0.19 mmol/L (*p* = 0.171) during the hypoglycaemic phase in HYPO and NORMO groups, respectively (Fig. 2B).

**Table 1:** Participants characteristics

	Control	Recurrent
Participants, n	16	16
Male/female, n	7/9	7/9
Age, y	44.3 ± 18.6	43.6 ± 17.9
HbA_1c_, mmol/mol	33.6 ± 3.5	35.4 ± 3.3
%	5.2 ± 0.3	5.4 ± 0.3
BMI, kg/m^2^	22.6 ± 2.8	23.6 ± 2.1

Data are presented as number (%), mean ± SD

In response to day 2 hypoglycaemia, all counterregulatory hormones increased significantly in the NORMO group.In the HYPO group, glucagon did not significantly increase and the responses of adrenaline and GH to hypoglycaemia were significantly blunted compared to the NORMO group. Symptom scores increased in response to day 2 hypoglycaemia, with no differences between HYPO and NORMO groups (Table 2).

**Table 2:** Hormone levels during day 1 and day 2 clamps

	Antecedent normoglycaemia		Antecedent hypoglycaemia	
	Day 1	Day 2	Day 1	Day 2
*Adrenaline (nmol/L)*				
Baseline	0.20 ± 0.12	0.23 ± 0.13	0.17 ± 0.10	0.14 ± 0.09^*#*^
Start hypo	0.18 ± 0.13	0.92 ± 1.08^†^	0.76 ± 0.62	0.23 ± 0.12^*#* †^
End of hypo	0.18 ± 0.14	2.68 ± 1.41*^†^	1.84 ± 1.16*	1.45 ± 1.24* ^*#*^
*Noradrenaline (nmol/L)*				
Baseline	1.44 ± 0.72	1.79 ± 0.58	1.67 ± 0.62	1.61 ± 0.68
Start hypo	1.35 ± 0.63	2.06 ± 0.68 ^†^	1.92 ± 0.62	1.67 ± 0.73
End of hypo	1.46 ± 0.75	2.51 ± 0.87*^†^	1.99 ± 0.82*	2.36 ± 1.43*
*Glucagon (ng/L)*				
Baseline	31.61 ± 10.49	30.86 ± 7.89	37.16 ± 25.13	35.99 ± 26.87
Start hypo		31.44 ± 12.69		33.77 ± 10.13
End of hypo	25.07 ± 5.99*	46.29 ± 19.69*^†^	62.82 ± 54.15*	48.28 ± 23.49
*Cortisol (umol/L)*				
Baseline	0.42 ± 0.10	0.38 ± 0.10	0.43 ± 0.13	0.37 ± 0.12^†^
Start hypo		0.25 ± 0.06		0.23 ± 0.11
End of hypo	0.28 ± 0.08	0.53 ± 0.11*^†^	0.51 ± 0.14*	0.48 ± 0.15*
*Growth hormone (mE/L)*				
Baseline	12.01 ± 13.47	8.37 ± 8.49	9.50 ± 11.08	5.45 ± 7.26^†^
Start hypo		4.96 ± 9.81		3.63 ± 5.27
End of hypo	7.06 ± 7.89	49.23 ± 28.19*^†^	35.75 ± 18.65*	28.34 ± 14.09*^#^

^*^p < 0.05 versus baseline

^*#*^*p* < 0.05 versus antecedent normoglycaemia Day 2

^†^*p* < 0.05 versus Day 1

**Fig. 3:**
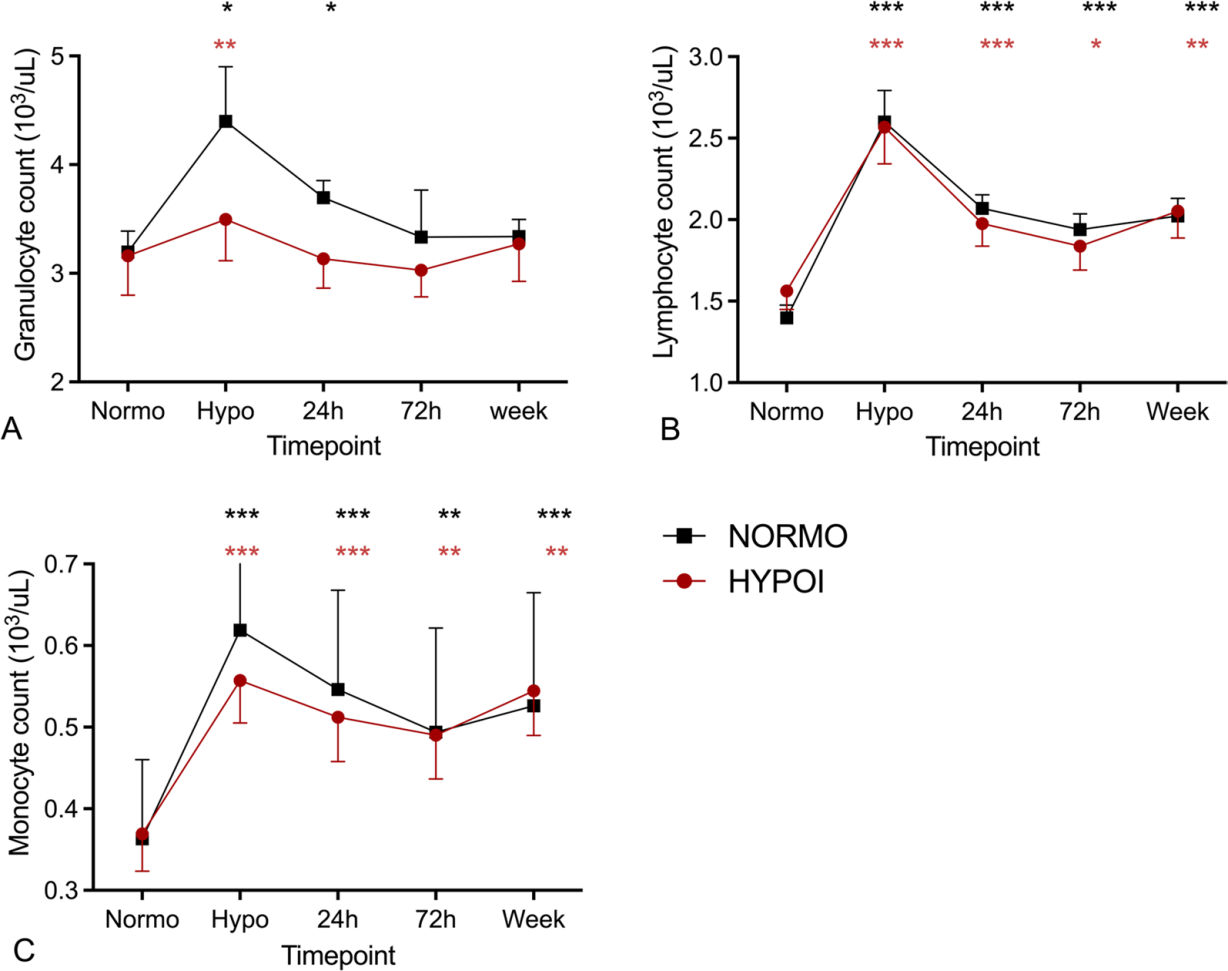
Counts (·10^3^/µL) of granulocytes (**A**), lymphocytes (**B**), and monocytes (**C**) after antecedent hypoglycaemia (HYPO) (red symbols) and antecedent normoglycaemia (NORMO) (black symbols). Data are presented as mean ± SEM, *p < 0.05, **p < 0.01 and ***p < 0.01 change versus normoglycaemia

Hypoglycaemia increased the number of granulocytes, lymphocytes and monocytes in both HYPO and NORMO groups (all* p* < 0.001), with no significant differences between the groups. Although not statistically significant, there was a trend towards a reduced increase in granulocyte number in the HYPO as compared to the NORMO group (1.55 ± 0.94 versus 2.67 ± 2.11·10^3^/µl, *p* = 0.072), which was due to one participant from the NORMO group with a much higher increase compared to all other participants..Granulocyte counts normalised 24 h after hypoglycaemia in the HYPO group, while it took 3 days to normalise in the NORMO group. The levels of lymphocytes and monocytes were also lower 24 h after the hypoglycaemic event but remained elevated compared to levels during normoglycemia for a week with no differences between HYPO and NORMO groups (both *p* < 0.001, Fig. 3).

**Fig. 4:**
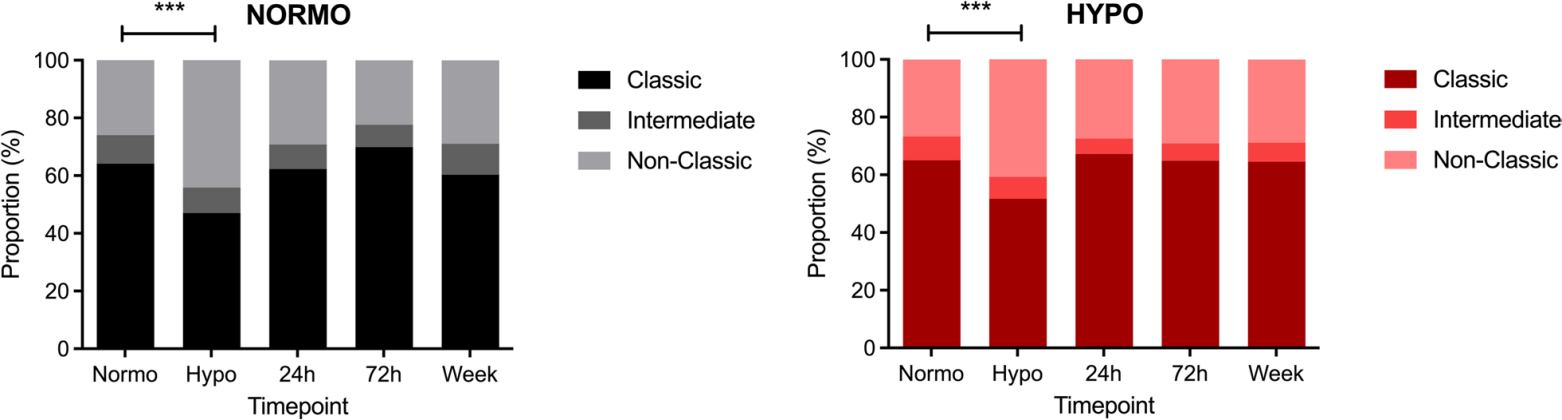
Proportion (%) of classical, intermediate and non-classical monocytes after antecedent normoglycaemia (top) and antecedent hypoglycaemia (bottom). ***p < 0.001 change versus normoglycaemia

Phenotypically, hypoglycaemia induced a shift from classical CD14^+^ monocytes towards more pro-inflammatory non-classical CD16^+^ monocytes in both groups (*p* < 0.001). This shift normalised 24 h after hypoglycaemia and was not significantly different between the two groups (Fig. 4).

**Fig. 5:**
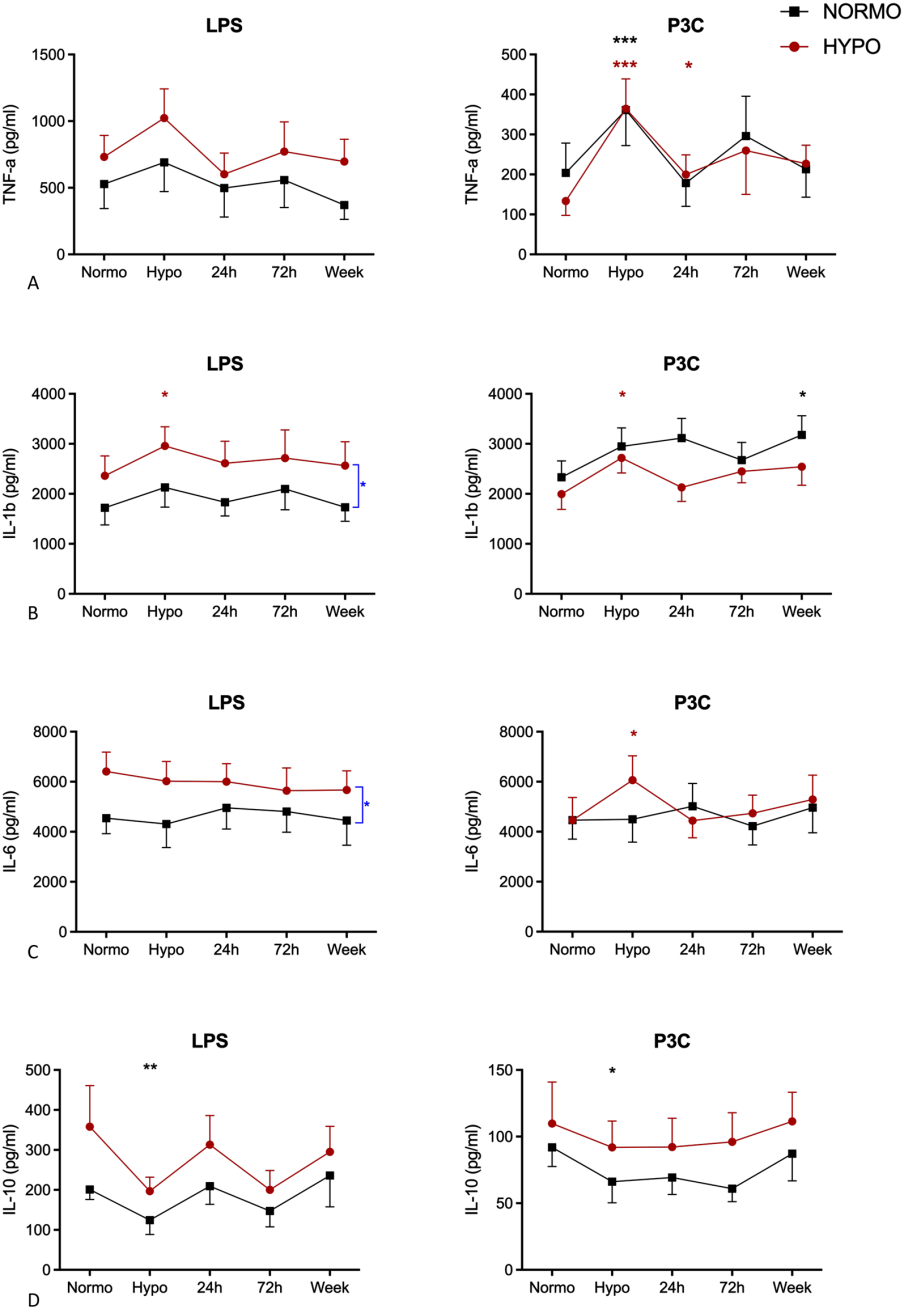
Ex vivo cytokine production of TNF-α (**A**), IL-1β (**B**), IL-6 (**C**) and IL-10 (**D**) upon LPS or P3C stimulation after antecedent hypoglycaemia (HYPO) (red symbols) and antecedent normoglycaemia (NORMO) (black symbols). Data presented as mean ± SEM, *p < 0.05, **p < 0.01 and ***p < 0.01 change versus normoglycaemia. Differences between groups are marked in blue *p < 0.05

Under normoglycaemic conditions on day 2, cytokine production appeared to be higher in the HYPO than in the NORMO group after ex vivo LPS stimulation, but not after P3C stimulation, which reached statistical significance for IL-1β and IL-6 (*p* < 0.05, Fig. 5). Hypoglycaemia caused a significant increase in LPS- or P3C-stimulated TNF-α and IL-1β production and a decrease in stimulated IL-10 production, which normalised after 24 h and none of which was modified by antecedent hypoglycaemia. Hypoglycaemia did not affect LPS- or P3C-stimulated IL-6 production in either group.

**Fig. 6:**
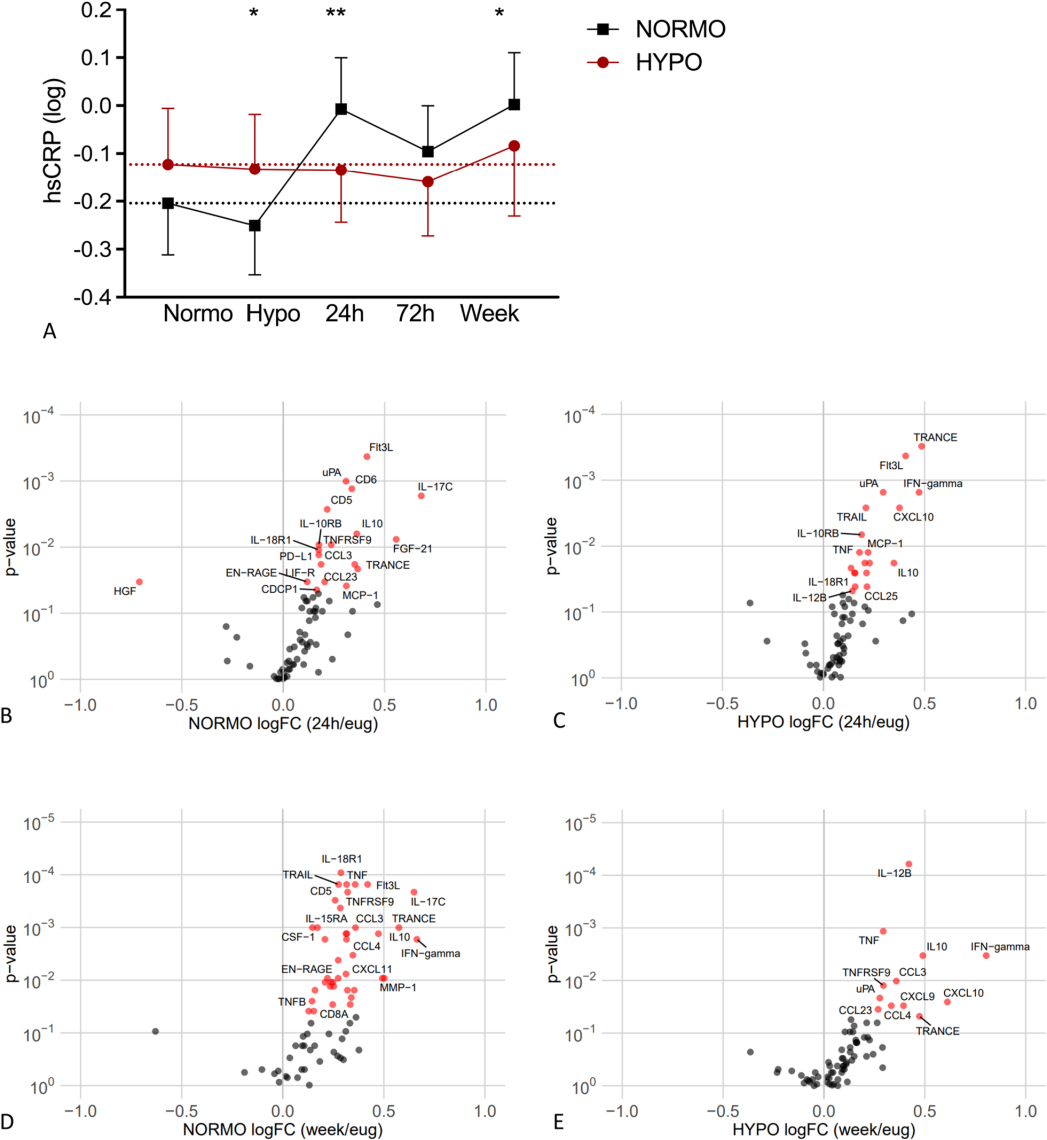
hs-CRP levels in plasma (**A**) over time after antecedent hypoglycaemia (HYPO) (red symbols) and antecedent normoglycaemia (NORMO) (black symbols). Data presented log2 fold change compared to normoglycaemia. Volcano plots of circulating inflammatory proteins in the antecedent hypoglycaemia (HYPO) and antecedent normoglycaemia (NORMO) groups after 1 (**B**, **C**) and 7 days (**D**, **E**) after hypoglycaemia compared to normoglycaemia. Proteins in red are significantly different compared to normoglycaemia (Wilcoxon paired test, *p* value < 0.05)

The pro-inflammatory protein hs-CRP was slightly higher in the HYPO compared to the NORMO group during day 2 normoglycaemia and remained unaltered in response to hypoglycaemia on day 2. In contrast, hs-CRP levels increased 24 h after hypoglycaemia in the NORMO group (*p* = 0.002) and remained elevated for a week (*p* = 0.044, Fig. 6A).

Both groups showed similar increases in other inflammatory proteins in response to hypoglycaemia (Fig. 6). After 24 h, inflammatory proteins that were upregulated included TRANCE, FIT3L, IFN-γ and IL-10RB, whereas after 1 week, these included TNF, IL-10, TNFRSF9 and TRANCE (Fig. 6B, C). There were no differences in responses of these inflammatory proteins between the HYPO and NORMO subgroups after 24 h, where 18 proteins were significantly higher in both groups. However, after 1 week, the number of proteins that were increased was lower in the HYPO (12 increased proteins) than in the NORMO group (39 increased proteins) (Fig. 6D, E).

**Fig. 7:**
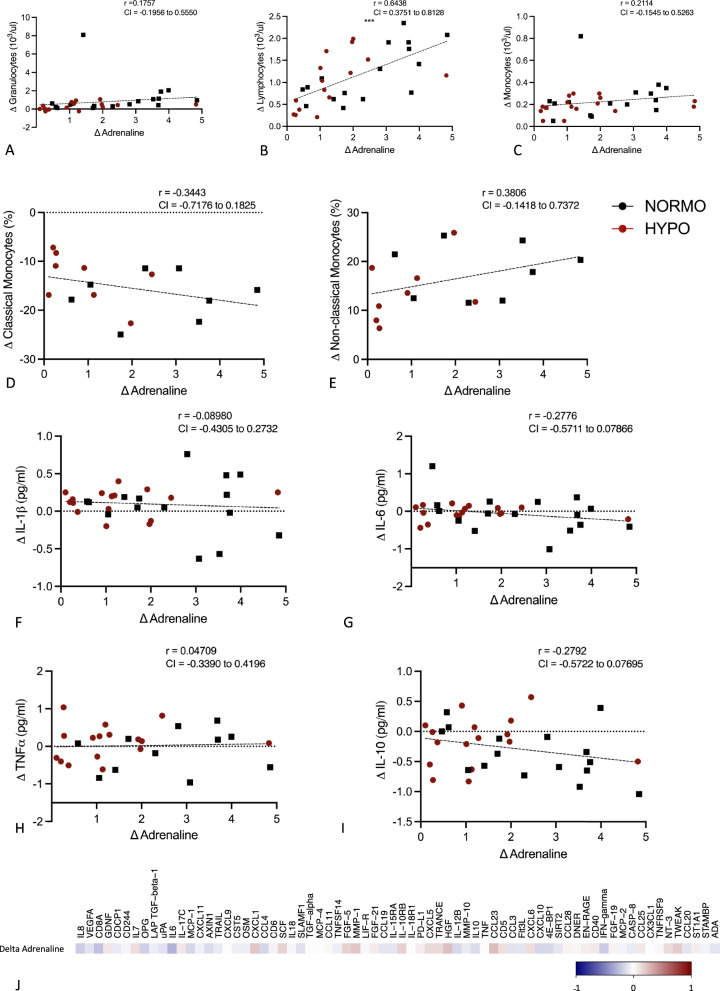
Scatter plots representing the correlation between the change in adrenaline during hypoglycaemia compared to normoglycaemia with the change in granulocytes (**A**), lymphocytes (**B**) and monocytes (**C**), monocyte phenotype (**D**, **E**), cytokine production (**F**–**I**) and inflammatory proteins (**J**) (Spearman test) during hypoglycaemia compared to normoglycaemia (Pearson test, unless otherwise stated), correlation coefficients (r) and confidence interval (CI) are depicted in the figures *p < 0.05, ** *p* < 0.01, ***p < 0.001)

**Fig. 8:**
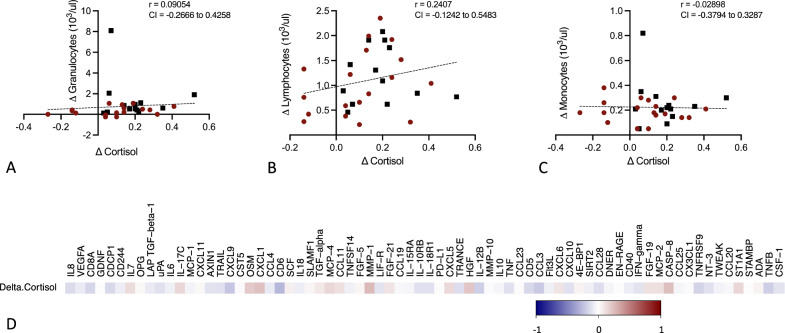
Scatter plots representing the correlation between the change in cortisol during hypoglycaemia compared to normoglycaemia with the change in granulocytes (**A**), lymphocytes (**B**) and monocytes (**C**), and inflammatory proteins (**D**) (Spearman test) during hypoglycaemia compared to normoglycaemia (Pearson test, unless otherwise stated), correlation coefficients (r) and confidence interval (CI) are depicted in the figures

The adrenaline response to hypoglycaemia was positively correlated with the increase of lymphocytes during hypoglycaemia in both groups combined (*p* < 0.001) (Fig. 7A). A similar positive correlation was seen between the adrenaline response and the increase in granulocytes and monocytes, but these did not reach statistical significance (Fig. 7B, C). The adrenaline response did not correlate with the change in monocyte phenotype, (Fig. 7D, E), the differences in cytokine production or the level of circulating inflammatory proteins during or after hypoglycaemia (Fig. 7F–J). Regarding the role of other counterregulatory hormones, none of the inflammatory responses to hypoglycaemia correlated with the cortisol or GH (data not shown) responses to hypoglycaemia in neither group (p < 0.05) (Fig. 8).

## Discussion

In this study, hypoglycaemia caused an acute and persistent pro-inflammatory effect, defined by changes in number, phenotype and function of monocytes combined with increased levels of various pro-inflammatory mediators. Although adrenaline responses were diminished after antecedent hypoglycaemia, there was no robust modifying effect of antecedent hypoglycaemia on these pro-inflammatory responses. Only the persistent response of circulating inflammatory markers seemed slightly decreased.

Our data are in line with earlier observations showing that hypoglycaemia acutely causes a range of pro-inflammatory responses [13–16], including increases in the number of immune cells [4, 5, 17], a phenotypical shift towards more pro-inflammatory non-classical monocytes and increases in cytokine production and release of pro-inflammatory proteins. We previously showed that many of these effects occur in both people with or without type 1 or type 2 diabetes, irrespective of level of glucose control or reported hypoglycaemic awareness [4, 5, 17]. The present study now extends these findings by showing that these pro-inflammatory effects are largely unaffected by recent, i.e. previous day, antecedent hypoglycaemia.

In line with a switch in monocyte phenotype, we also found a switch in monocyte function in that ex vivo pro-inflammatory cytokine production increased during hypoglycaemia, whereas ex vivo anti-inflammatory production decreased [17]. Somewhat surprisingly, we observed a trend towards increased rather than decreased levels of cytokine production after antecedent hypoglycaemia following ex vivo stimulation with LPS, again arguing against an attenuating effect of antecedent hypoglycaemia on the immune system. This suggests that hypoglycaemia causes changes occur in pathogen-specific signalling pathways which potentially affect the expression of pattern recognition receptors on the cell surface or their downstream effectors.

Interestingly, we found an attenuating effect of antecedent hypoglycaemia on the hs-CRP response to next-day hypoglycaemia. However, this attenuated response was not directly related to the suppressed adrenaline response in participants exposed to prior hypoglycaemia. Therefore, it could be that adrenaline is not the origin of the pro-inflammatory response but a marker of the response.

When looking at other circulating markers of inflammation, we found a sustained increase lasting up to 1 week of various circulating inflammatory proteins, including IL-12B. This cytokine stimulates T and NK-cells to produce IFN-γ, which was also found to increase in response to hypoglycaemia [18]. IFN-γ in turn activates macrophages, which then produce TNF-α [19]. In addition, CXCL9, CCL23, CCL3 and CLL4 increased in response to hypoglycaemia, which can recruit immune cells, thus potentially contributing to the persistently elevated levels of circulating white blood cells [20]. Overall, these observations indicate that hypoglycaemia activates both the innate and adaptive immune system and that the activation lasts for 1 week. Interestingly, we found fewer proteins to be elevated in the HYPO group, which suggests that antecedent hypoglycaemia may have a modest inhibiting effect on the immune activation following hypoglycaemia. This has also been seen in animal models where in both mice with and without type 1 diabetes, recurrent hypoglycaemic amplified inflammatory markers in hippocampal homogenates [21].

The adrenaline response has been suggested to be crucial in starting the inflammatory response following hypoglycaemia. Exposure to adrenaline has been associated with inflammatory changes in granulocytes and monocytes in vitro and in vivo [22]. In agreement with previous observations [23], we observed a diminished adrenaline response in the HYPO group compared to the NORMO group. We found a positive correlation between the adrenaline response during hypoglycaemia and increase in lymphocyte numbers in the total study population and a similar trend for numbers of granulocytes and monocytes. The increase in pro-inflammatory cytokines following hypoglycaemia was independent of the adrenaline response and the exposure to antecedent hypoglycaemia. This contrasts with previous research that found an immunosuppressive effect of adrenaline on ex vivo cytokine production [22]. Overall, our findings suggests that the pro-inflammatory effects of hypoglycaemia, as investigated here, are not solely driven by the adrenaline response. It is also possible that the adrenaline response that we observed in the antecedent hypoglycaemia group, albeit attenuated, was still sufficient in initiating the immune response. Other hormones like cortisol can be involved in the pro-inflammatory response [3]. The lack of correlation between cortisol and the inflammatory readouts argues against this option. Another explanation for the modest role of adrenaline found in this study could be a lack of power, as the main objective of this study was not to determine the role of adrenaline in inflammation following hypoglycaemia, fow which further research is needed.

Apart from a somewhat lower number of proteins activated by hypoglycaemia after antecedent events, overall there was largely no adaptation (at least not after two episodes) regarding the inflammatory response to hypoglycaemia. This is in contrast with the attenuating effect of recurrent hypoglycaemia on hormone responses to subsequent hypoglycaemia. Previously, an attenuated inflammatory response to hypoglycaemia was found in people with impaired awareness of hypoglycaemia [4]. One could speculate that this attenuated response might protect against harmful effects of subsequent hypoglycaemia. However, a blunted adrenaline response to hypoglycaemia following recurrent episodes of hypoglycaemia did not protect against markers of vascular dysfunction [2]. Furthermore, persistent prothrombotic effects have been reported following recurrent hypoglycaemia [13]. These findings, along with ours, suggest that the inflammatory response following hypoglycaemia occurred largely irrespective of prior exposure to hypoglycaemia, level of hypoglycaemic awareness and glycaemic control.

Our study has limitations. The induced hypoglycaemia events were highly controlled and maintained for a certain duration with the hyperinsulinaemic glucose clamp technique in people without diabetes, whereby the hypoglycaemic events may differ in depth, duration and number from spontaneous hypoglycaemia in daily life in people with diabetes treated with insulin. However, this method ensured that all participants underwent an identical hypoglycaemic event, so that we were able to compare the results between the two groups. Also, we cannot exclude an attenuating effect of more than two hypoglycaemic events on the inflammatory response, although this still does not explain the discrepancy with adrenaline. Another limitation is that the rather high doses of insulin, such as those used for the clamps, can reduce pro-inflammatory responses [2]. However, despite any potential anti-inflammatory effects of insulin, we were still able to observe pro-inflammatory effects of hypoglycaemia.

Our study has also strengths. The two investigated groups were well matched for age, sex and BMI. In addition, we provided a comprehensive assessment of the inflammatory profile, including leukocyte cell counts, phenotype of monocytes, function of monocytes and circulating inflammatory proteins. Finally, unlike most previous studies on the consequences of hypoglycaemia on inflammation, our investigations continued up to a week after the hypoglycaemic event.

In conclusion, our study provides evidence that the inflammatory response to hypoglycaemia occurs largely, but not completely, independent of prior exposure to hypoglycaemia. Future research is needed to elaborate further on the mechanisms that underly the observed inflammatory effect, and the potential role of the inflammatory response on the development of cardiovascular complications.

### Supplementary Information

**Additional file 1**. Supplement 1: in-and exclusions criteria.

## Data Availability

The data that support the findings of this study are not openly available due to reasons of sensitivity and are available from the corresponding author upon reasonable request.

## Abbreviations

BMI: Body mass index
CV: Coefficient of variation
*C. albicans*: *Candida albicans*
ECAR: Extracellular acidification rate
HbA_1c_: Glycated haemoglobin
hs-CRP: High sensitive C-reactive protein
IL: Interleukin
LPS: Lipopolysaccharide
MACS: Magnetic activated cell sorting
*mTBC*: *Mycobacterium tuberculosis*
OCR: Oxygen consumption rate
P3C: Pam3Cys
PBMC: Peripheral blood mononuclear cells
TNF: Tumour necrosis factor
*S. aureus*: *Staphylococcus aureus*
