# Supplementary material for: The impact of prior exposure to hypoglycaemia on the inflammatory response to a subsequent hypoglycaemic episode
Source: Cardiovasc Diabetol. 2024 Feb 8;23:55. doi: 10.1186/s12933-023-02095-w (PMC10854178; doi:10.1186/s12933-023-02095-w)

**Electronic supplementary materials**

*ESM methods*

Inclusion criteria - overall

- Ability to provide written informed consent
- Must be able to speak and read Danish (for Hillerød-site) and Dutch (for Nijmegen-site)
- BMI: 19-40 kg/m^2^
- Age ≥18 years, ≤ 80 years
- Blood pressure: < 140/90 mmHg
- HbA_1c_ < 42 mmol/mol (6%) and age ≥ 18 years, ≤ 80 years

Exclusion criteria

- Presence of any medical condition that might interfere with the study protocol, such as brain injuries, epilepsy, a major cardiovascular disease event or anxiety disorders
- Use of any medication, except for oral contraceptives, stable dose of thyroxine
- Pregnancy or breastfeeding or unwillingness to undertake measures for birth control
- Any infection in the three months before screening
- Vaccination in the three months before screening

**ESM Table 1** Symptom response

|  | antecedent normoglycaemia | | | Antecedent hypoglycaemia | | |
| --- | --- | --- | --- | --- | --- | --- |
|  | Day 1  Clamp 1 | Day 1  Clamp 2 | Day 2  Clamp 3 | Day 1  Clamp 1 | Day 1  Clamp 2 | Day 2  Clamp 3 |
| Total |  |  |  |  |  |  |
| Baseline | 24.56 ± 7.87 | 23.19 ± 6.27 | 20.38 ± 2.09 | 23.63 ± 4.75 | 26.00 ± 4.05 | 21.44 ± 2.71 |
| Start Hypo | 23.38 ± 5.44 | 23.56 ± 6.11 | 24.19 ± 4.59 | 26.94 ± 7.39 | 28.63 ± 7.21 | 27.63 ± 0.10 |
| End of hypo | 24.25 ± 6.57 | 23.69 ± 4.88 | 38.56 ± 7.72* | 36.33 ± 12.49* | 36.75 ± 13.77* | 36.56 ± 15.78* |
| Neuroglycopenic |  |  |  |  |  |  |
| Baseline | 12.25 ± 4.55 | 12.44 ± 4.68 | 10.63 ± 1.63 | 12.75 ± 3.79 | 14.00 ± 2.90 | 11.13 ± 1.93 |
| Start Hypo | 11.69 ± 3.36 | 12.75 ± 4.68 | 12.88 ± 3.34 | 14.75 ± 5.07 | 15.13 ± 4.60 | 15.31 ± 6.41 |
| End of hypo | 12.94 ± 5.11 | 12.69 ± 3.93 | 20.56 ± 5.62* | 20.00 ± 8.14* | 18.88 ± 8.16* | 20.31 ± 9.33* |
| Autonomic |  |  |  |  |  |  |
| Baseline | 9.75 ± 2.32 | 8.50 ± 1.59 | 7.69 ± 0.79 | 8.88 ± 1.67 | 9.27 ± 1.28 | 8.25 ± 1.18 |
| Start Hypo | 9.56 ± 2.19 | 8.38 ± 1.45 | 9.13 ± 2.55 | 9.94 ± 2.41 | 10.56 ± 2.66 | 10.06 ± 3.55 |
| End of hypo | 9.06 ± 1.53 | 8.56 ± 1.41 | 15.50 ± 4.79* | 13.73 ± 4.77* | 14.81 ± 5.37* | 13.94 ± 6.10* |
| General Malaise |  |  |  |  |  |  |
| Baseline | 2.56 ± 1.31 | 2.25 ± 0.68 | 2.06 ± 0.25 | 2.00 ± 0.00 | 2.73 ± 0.80 | 2.06 ± 0.25 |
| Start Hypo | 2.13 ± 0.34 | 2.44 ± 0.81 | 2.19 ± 0.40 | 2.25 ± 0.58 | 2.94 ± 1.00 | 2.25 ± 0.58 |
| End of hypo | 2.25 ± 0.58 | 2.44 ± 0.73 | 2.50 ± 1.26* | 2.60 ± 0.83* | 3.06 ± 1.34* | 2.31 ± 0.70* |

******p* < 0.05 versus baseline

*# p* < 0.05 versus antecedent normoglycaemia Day 2

† *p* < 0.05 versus Day 1

**ESM Figure 3** Gating strategy of the flow cytometry analysis.


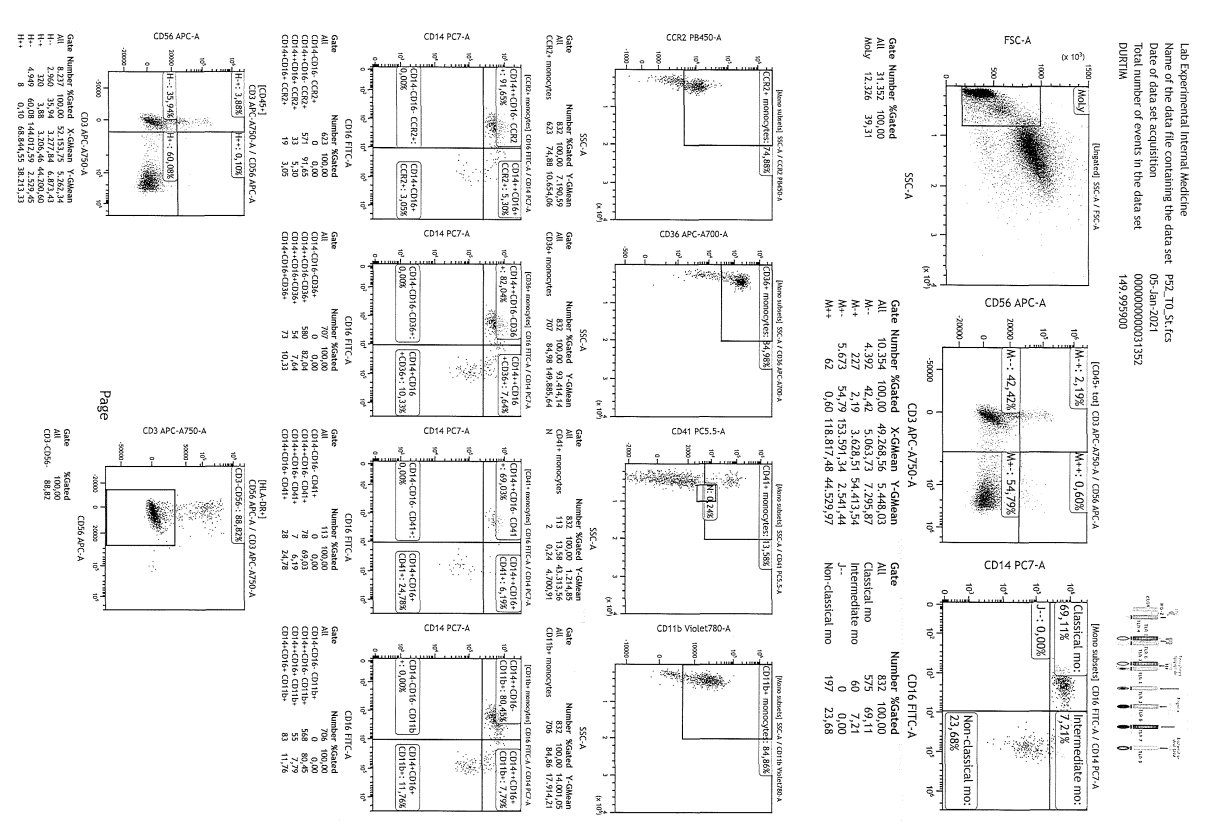

Supplement: Supplementary file 1 — Additional file 1. Supplement 1: in-and exclusions criteria. [file 12933_2023_2095_MOESM1_ESM.docx]
